# Clinical significance of migration and invasion inhibitor protein expression in non-small-cell lung cancer

**DOI:** 10.3892/ol.2014.2526

**Published:** 2014-09-12

**Authors:** XINHUA WANG, HONGLING LIU, XIAOYU WANG, YUZHI AN

**Affiliations:** 1 Department of Oncology, The First Affiliated Hospital of Liaoning Medical University, Jinzhou, Liaoning 121000, P.R. China; 2 Department of Respiratory Internal Medicine, Qingzhou People’s Hospital, Qinzhou, Shandong 262500, P.R. China

**Keywords:** non-small-cell lung cancer, migration and invasion inhibitor protein, real-time polymerase chain reaction, immunohistochemistry, prognosis

## Abstract

Migration and invasion inhibitor protein (MIIP) was initially identified in a yeast two-hybrid screen. Recently, MIIP has emerged as a key protein in regulating cell migration and invasion. However, the MIIP expression profile in non-small-cell lung cancer (NSCLC) has not been analyzed. In the present study, MIIP mRNA expression levels were evaluated using the SYBR Green quantitative real-time polymerase chain reaction method in 37 NSCLC specimens and matched normal tissue samples. MIIP protein expression in a further 94 NSCLC specimens was examined with immunohistochemistry. Patient survival data were collected retrospectively, and the association between MIIP protein expression and the five-year overall survival rate was evaluated. The results revealed that MIIP mRNA and protein expression were downregulated in cancer tissues, as compared with the matched normal tissues. MIIP expression levels were significantly associated with pathology and tumor stage, with reduced MIIP mRNA expression levels detected in advanced tumor stage samples. Furthermore, patients with MIIP-positive protein expression had an improved prognosis as compared with those patients with MIIP-negative protein expression, with five-year survival rates of 41.7 and 22.4%, respectively (Kaplan-Meier, log-rank, P=0.028). A significant association between MIIP protein expression and improved prognosis was also demonstrated using univariate and multivariate analyses (P=0.033 and P=0.040, respectively). These results suggest that MIIP may have a potential role in the pathogenesis of NSCLC and also confirm that MIIP is a putative tumor-suppressor gene. Therefore, MIIP may be identified as a functional genetic marker of NSCLC development and prognosis, and may be an attractive therapeutic target for the treatment of lung cancer.

## Introduction

Lung cancer remains the leading cause of cancer-related mortality worldwide, and is particularly prevalent in males (1). In China, the prevalence of lung cancer has grown rapidly over the past five years (2). The main types of lung caner are small-cell lung carcinoma (SCLC) and non-small-cell lung carcinoma (NSCLC). Among all lung cancer cases, almost 85% are NSCLC (3), which is further categorized into two predominant types: Non-squamous carcinoma (including adenocarcinoma, large-cell carcinoma and other cell types) and squamous cell carcinoma. Despite recent advances in the diagnosis and treatment of lung cancer, the improvement in survival rate has only been modest, with an overall five-year survival rate of <15% (4). Cancer cell invasion, identified in 30–40% of NSCLC patients with poor prognosis, is a critical determinant of survival. Therefore, elucidating the potential biological markers of metastasis is urgently required for guidance on postoperative surveillance and therapeutic decisions.

The migration and invasion inhibitor protein (MIIP), also known as invasion inhibitory protein 45 (IIp45), is a recently characterized putative tumor suppressor gene in glioma (5). MIIP was initially identified in a yeast two-hybrid screen for proteins that interact with protein insulin-like growth factor binding protein 2 (IGFBP2) (6). MIIP is located on chromosome 1p36.22 and recent genome analysis has determined that MIIP contains 10 exons that span 12.6 kb genomic DNA. The full-length transcript contains 1,588 bp. The MIIP protein is comprised of 388 amino acids and has a predicted molecular weight of 43 kDa. MIIP is a hydrophilic protein and contains three segments of low compositional complexity domains and an arginine-glycine-aspartate motif (7). The 1p36 region is deleted in a number of types of cancer, including neuroblastoma, breast cancer, colon and rectum cancer, and prostate cancer, as well as lung cancer (8–12). Recently, MIIP has emerged as a key protein in regulating cell migration, cell invasion and the mitosis checkpoint and, thus, may exert a critical role in cancer physiology (6,13). However, MIIP expression profiles have, to the best of our knowledge, not been described in NSCLC. The present study aimed to detect MIIP expression using real-time polymerase chain reaction (PCR) and immunohistochemistry (IHC) methods in resected tissue samples and formalin-fixed paraffin sections from patients with NSCLC. Whether MIIP expression correlated with pathological and clinical features was also evaluated. In addition, the association between MIIP expression and the five-year overall survival rate was analyzed.

## Materials and methods

### Study population and samples

Fresh tumor tissues and matched normal tissues from 37 NSCLC patients (diagnosed in 2011 and 2012) were immediately transferred to liquid nitrogen and stored at −80°C for subsequent real-time PCR analysis, and 94 formalin-fixed paraffin-embedded samples of NSCLC tissues (patients diagnosed in 2007 and 2008) were obtained for immunohistochemical analysis. All patients were diagnosed in the First Affiliated Hospital of Liaoning Medical University (Jinzhou, China) and samples from the patients were stored in the pathology archive. Permission from patients was obtained prior to specimen collection. None of the patients had received chemotherapy, radiotherapy or immunotherapy prior to surgery. The study was approved by the Ethics Committee of the First Affiliated Hospital of Liaoning Medical University. Histopathological evaluation was conducted independently by two pathologists. All cases were classified according to the World Health Organization revised proposal for histological types of lung and pleural tumors (14). TNM patient evaluation was performed according to the criteria indicated in the staging procedures of the International Association for the Study of Lung Cancer (15). The patients enrolled in the IHC analysis were followed-up for five years to determine survival time, which was defined as the time period between the date of surgery on the primary tumor and when the patient succumbed to disease or the date of the final follow-up. Patient survival times were individually provided by family members by telephone. The clinicopathological data are summarized in Table I.

### Real-time PCR

Total RNA was isolated from the frozen NSCLC and matched normal tissues using the TRIzol RNA kit (Invitrogen, Carlsbad, CA, USA) and quantified using an Eppendorf Biophotometer (Eppendorf, Hamburg, Germany). Reverse transcription was conducted using a PrimeScript™ RT reagent kit with gDNA Eraser (Takara Bio, Inc., Shiga, Japan). Under the thermal conditions recommended by the manufacturer, 2 μg total RNA was transcribed to cDNA in a 20 μl reaction using random hexamers. The following primers were used for quantification of MIIP mRNA expression levels: Forward, 5′-GGT CCA TCC TGG CTC AAC AGA-3′ and reverse, 5′-GCA ATC CAG TCA TAG CCC AGG TA-3′. The length of the PCR product was 118 bp. Real-time PCR was performed using Mastercycler^®^ ep realplex (Eppendorf, Hamburg, Germany) and the SYBR^®^ Premix Ex Taq^™^ kit (Takara Bio., Inc.). GAPDH served as a control with the following primers used: Forward, 5′-GCA CCG TCA AGG CTG AGA AC-3′ and reverse, 5′-TGG TGA AGA CGC CAG TGG A-3′, with the length of PCR product at 138 bp. PCR was run for 40 cycles with 5 sec per 95°C denaturation, 30 sec/55°C annealing and 30 sec/72°C elongation. To verify the accuracy of the amplification, a melting curve analysis was conducted subsequent to amplification. In addition, the PCR products were verified by electrophoretic analysis on a 3% agarose gel. To determine the relative expression levels of MIIP, the comparative Ct method was used. The Ct value of the target gene was normalized to that of the endogenous reference [ΔCT = CT(target) − CT(GAPDH)] and compared with a calibrator [ΔΔCT = ΔCT(target) − ΔCT (calibrator)]. The relative expression levels of the target gene were calculated via the 2^−ΔΔCT^ method.

### Immunohistochemical staining for MIIP

MIIP protein expression on the formalin-fixed paraffin sections was determined by IHC. Briefly, 5-μm tissue sections were dewaxed in xylene, rehydrated and incubated in 0.3% (v/v) hydrogen peroxide in 0.01 M phosphate-buffered saline (PBS; pH 7.6) for 20 min to inactivate endogenous peroxidase. Antigen retrieval was performed by heating the sections with 10 mm citrate buffer (pH 6.0) for 15 min in a microwave. The sections were incubated with rabbit anti-human polyclonal primary anti-MIIP antibody (1:300; Sigma-Aldrich, St. Louis, MO, USA) at 4°C overnight, washed in PBS three times for 15 min and incubated with horseradish peroxidase/Fab Polymer-conjugated mouse anti-rabbit monoclonal secondary antibody (Zhongshan Biotechnology Inc., China) for 30 min at room temperature. Thereafter, the antibody was revealed by incubation with diaminobenzidine at room temperature for 1 min. The sections were counterstained with hematoxylin, dehydrated and mounted. The MIIP immunoreactivity level was classified using the proportion of positive cells: 0, <5% positive cells; 1+, 5–30% positive cells; 2+, 31–50% positive cells; and 3+, >50% positive cells. The intensity of MIIP expression was scored as follows: 0, negative to weak; 1, moderate; and 2, strong. The final staining score was the sum of the intensity and the percentage of positive cells scores. A score of ≤1 was applied as a cut-off point for loss of MIIP expression.

### Statistical analysis

The real-time PCR values are presented as mean ± standard error of the mean, and MIIP protein expression was dichotomized as either ‘negative’ or ‘positive’, according to the criteria described above. A two-sample t-test for independent samples was used for continuous variables. The correlation between MIIP expression and clinicopathological characteristics was then analyzed using the χ^2^-test. One-way analysis of variance was used to compare the means of two or more independent groups. The survival curves for patient with MIIP-positive and -negative tumors were plotted using the Kaplan-Meier method and the log-rank test was used to assess the statistical difference between the groups. Univariate and multivariate analyses were conducted using the Cox proportional-hazards regression model, and the results are expressed as hazard ratios with 95% confidence intervals. Two-tailed P<0.05 was considered to indicate a statistically significant difference. All analyses were performed using SPSS for Windows, version 13.0 (SPSS, Inc., Chicago, IL, USA).

## Results

### Expression levels of MIIP mRNA in NSCLC and adjacent normal lung samples

**Figure 1:**
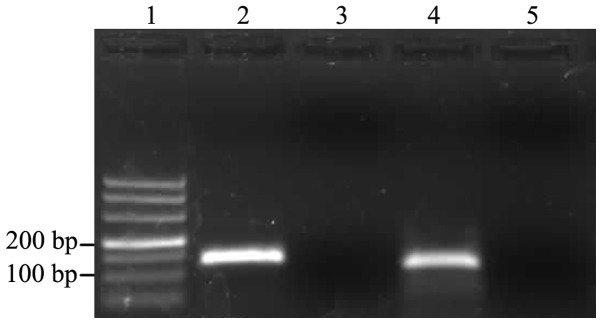
Gel electrophoresis analysis of real-time polymerase chain reaction products. Lane 1, 500 bp molecular size marker; lane 2, GAPDH; lane 3, no template control for GAPDH; lane 4; migration and invasion inhibitor protein (MIIP); lane 5; No template control for MIIP.

The MIIP mRNA expression levels in cancer tissues and matched normal tissues from 37 NSCLC patients were examined using the ΔΔCT method. Melting curve analysis confirmed the specific amplification of the target and reference genes. Furthermore, gel electrophoresis analysis of the amplification products revealed single bands of the expected sizes for MIIP (118 bp) and GAPDH (138 bp) (Fig. 1). The results demonstrated that MIIP expression was downregulated in the cancer tissues. The average MIIP mRNA expression level in the 37 cancer tissue samples was 0.1867±0.0217.

### Correlation between MIIP mRNA expression levels and various clinicopathological parameters

**Figure 2:**
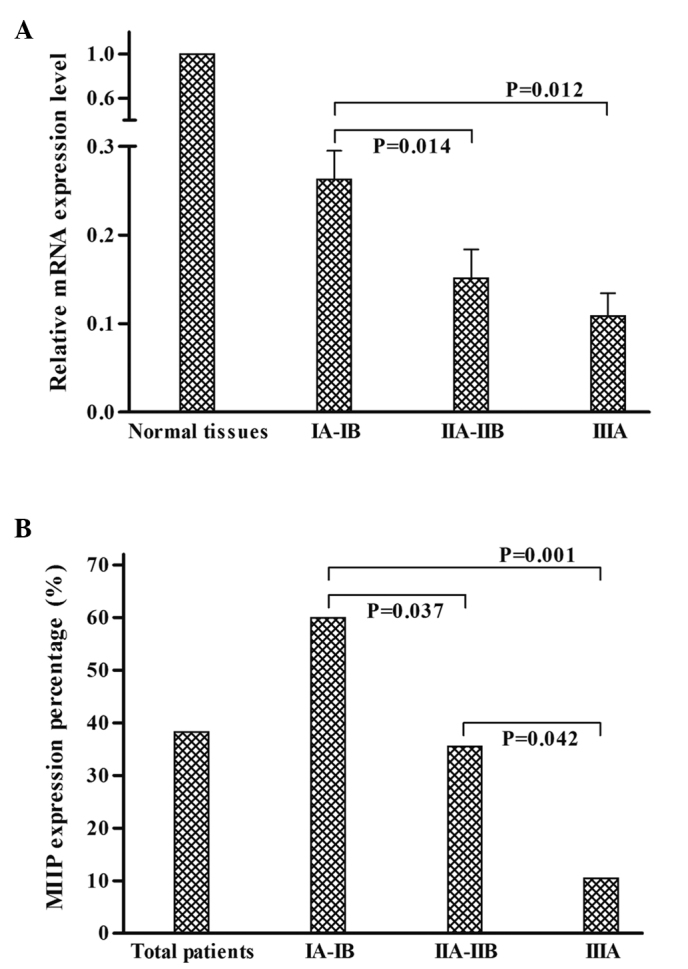
Migration and invasion inhibitor protein (MIIP) expression within tumor stage groups. (A) The relative MIIP mRNA expression levels in normal tissues and tumor tissues at different stages; (B) the percentage of positive MIIP protein expression in all patients and in patients with tumors at different stages.

The association between MIIP mRNA expression levels in the cancer tissues and various clinicopathological parameters was further analyzed. The results revealed that the MIIP mRNA expression levels in adenocarcinoma were significantly higher than those in squamous cell carcinoma (P=0.002). A statistically significant correlation was also observed between MIIP mRNA expression and tumor stage (P=0.014), with reduced MIIP mRNA expression levels in advanced tumor stage samples, as compared with specimens from tumors at the lower stages (Fig. 2A). However, no significant correlations were observed between MIIP expression levels and gender, age or differentiation status (all P>0.05), as shown in Table II.

### Correlation between MIIP protein expression and various clinicopathological parameters

**Figure 3:**
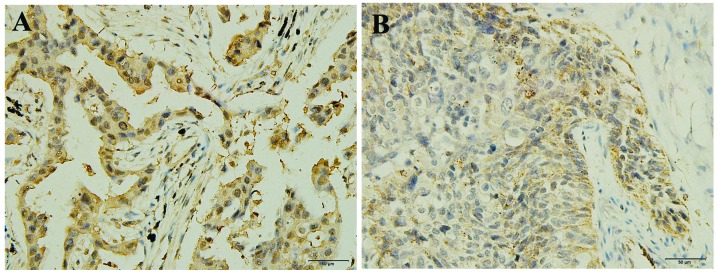
Examples of migration and invasion inhibitor protein immunostaining in non-small cell lung carcinoma samples. (A) Adenocarcinoma; and (B) squamous cell carcinoma.

Immunohistochemical staining of MIIP in the cancer tissue sections was conducted. Immunoreactivity for the MIIP antibody was predominantly identified in the cytoplasm of cancer cells with marginal immunoreactivity in the nucleus (Fig. 3). Among the 94 tissue samples, MIIP protein expression was positive in 36 (38.3%) and negative in 58 (61.7%) cases. The results revealed that MIIP protein expression was downregulated in cancer tissues, a finding in concordance with the mRNA expression result. The association between MIIP expression in NSCLC tissues and various clinicopathological parameters was also analyzed. Analysis of MIIP expression revealed that expression was significantly associated with pathology and tumor staging (P=0.014 and P=0.002, respectively), but not with gender, age or NSCLC differentiation status (all P>0.05), as shown in Table III. To determine whether the downregulation of MIIP protein expression was correlated with disease progression, the staining degrees within tumor staging groups were compared. The results demonstrated that the positive percentage of MIIP protein expression was significantly reduced in advanced tumor stage samples, as compared with specimens from less advanced tumors (Fig. 2B; Table III).

### Survival analysis

**Figure 4:**
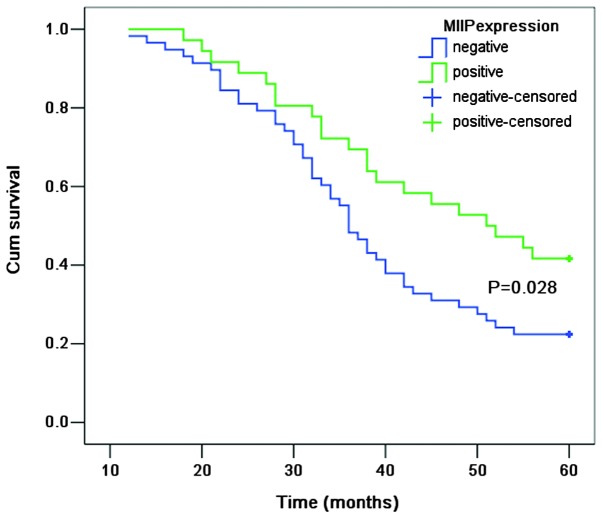
Kaplan-Meier analysis in patients with non-small-cell lung cancer according to migration and invasion inhibitor protein (MIIP) expression. Log-rank test, P=0.028.

The total follow-up time period for the patients who were alive at the time of analysis was five years. A total of 66 (70.2%) of the 94 patients succumbed to disease during the follow-up period. The five-year survival rate within the patient population was 29.8%. With regard to MIIP expression status, the five-year survival rate for patients who were MIIP-positive was 41.7% as compared with 22.4% for patients who were MIIP-negative. According to the Kaplan-Meier survival analysis, the overall survival rate curve demonstrated statistically significant differences between NSCLC patients with and without MIIP expression (P=0.028; Fig. 4). Cox proportional-hazards regression model was employed to perform univariate and multivariate analysis of survival. The results revealed that positive MIIP expression is an independent and significant factor associated with improved five-year survival. The detailed results are shown in Table IV.

## Discussion

Cell motility is important for normal tissue development and remodeling as well as for pathological conditions, such as cancer invasion and metastasis (16). Lung cancer is the leading cause of cancer-related mortality. Approximately 90% of all cancer mortality is the result of metastases, rather than the primary tumor (17). Thus, further understanding of the underlying pathways and molecular mechanisms of lung cancer metastasis is required to develop novel therapeutic approaches. MIIP has emerged as a key protein in regulating cell migration and invasion (6). However, the importance of MIIP in NSCLC is unknown. The present study aimed to examine the expression of MIIP in NSCLC with respect to prognosis. The results of real-time PCR and immunohistochemical staining clearly demonstrated that MIIP expression was downregulated in cancer tissues, as compared with matched normal tissues, a finding consistent with the results previously reported in a study analyzing glioblastoma multiforme (6). In addition, in the present study, MIIP expression levels were significantly correlated with pathology and tumor staging, suggesting a potential role for MIIP protein in the pathogenesis of NSCLC. Furthermore, the prognostic significance of MIIP expression in formalin-fixed paraffin-embedded tissues was examined. Notably, patients with positive MIIP expression had a significantly improved overall survival rate (P=0.028). A significant association was also observed between positive MIIP expression and improved prognosis using univariate and multivariate analyses. These results suggest that MIIP may be a functional genetic marker of NSCLC development and prognosis, and that MIIP may be an attractive therapeutic target in the treatment of lung cancer.

The MIIP protein has been previously demonstrated to bind to a product of an oncogene, IGFBP-2, which is commonly upregulated in the advanced stages of cancer, and to inhibit the migration- and invasion-enhancing functions of IGFBP-2 (6). Studies have revealed that MIIP expression levels were reduced in glioblastoma multiforme and that IIp45 expression levels were low in advanced glioma (6,18). In the present study, similar results were observed in NSCLC. The data demonstrate that MIIP expression levels were significantly correlated with tumor staging, and reduced MIIP mRNA and protein expression levels were detected in advanced tumor stage samples. The MIIP protein has been previously observed to inhibit glioma cell migration and invasion *in vitro* and *in vivo*, and a xenograft model study revealed that tumors formed from MIIP-expressing cells were also less invasive as compared with the controls (6). Another study reported that MIIP mediates histone deacetylase 6 activity, which regulates microtubule dynamics/cytoskeletal structure, increases cell migration and increases acetylated alpha-tubulin (18). In addition to an antimigration and -invasion function, MIIP has also been shown to be involved in mitosis (13), as elevated MIIP expression levels inhibited glioma cell colony formation and cell growth *in vitro*, and MIIP expression in a glial-specific mouse model suppressed glioma development and progression (13). Epidemiology studies have provided further evidence that MIIP is important in cancer development. Several MIIP single nucleotide polymorphisms (SNPs) were recently evaluated in a molecular epidemiology study involving 1,524 breast cancer patients and 1,592 healthy females, which found that the rs2295283 (K167E) SNP was not only associated with breast cancer risk but also with various tumor phenotypes in cancer patients (19). All these results from recent studies raise the possibility that MIIP is a putative tumor-suppressor gene, critical in cancer physiology.

In conclusion, the present study has revealed that MIIP was downregulated in NSCLC tissues. According to the survival analysis, MIIP expression was an independent prognostic factor associated with improved survival. All data suggest that MIIP is a tumor suppressor gene, with a critical role in NSCLC physiology. Since the present study was a preliminary investigation, the number of patients with NSCLC was relatively small (94 patients). Further experiments are required in order to investigate the exact mechanism of MIIP involvement in lung carcinogenesis. The significant association with survival rate observed in the present study requires confirmation in additional patient cohorts.

## Figures and Tables

**Table I:** Clinicopathological features of the NSCLC patients.

	Total no. of patients (n)	
	
Clinicopathological feature	Real-time PCR analysis (n=37)	Immunohistochemical analysis (n=94)
Gender		
Male	19	54
Female	18	40
Age (years)		
<60	17	43
≥60	20	51
Pathology		
Adenocarcinoma	19	45
Squamous cell carcinoma	18	49
Differentiation status		
Well	14	29
Moderate	11	43
Poor	12	22
Tumor staging		
IA–IB	14	30
IIA–IIB	17	45
IIIA	6	19

NSCLC, non-small cell lung cancer; PCR, polymerase chain reaction.

**Table II:** Correlation between MIIP mRNA expression and clinicopathological features.

Clinicopathological feature	No. of patients	MIIP expression level (mean ± SE)	P-value
Gender			0.832
Male	19	0.1822±0.0306	
Female	18	0.1916±0.0315	
Age (years)			0.265
<60	17	0.1642±0.0281	
≥60	20	0.2133±0.0334	
Pathology			0.002
Adenocarcinoma	19	0.2488±0.0282	
Squamous cell carcinoma	18	0.1213±0.0257	
Differentiation status			0.435
Well	14	0.2117±0.0356	
Moderate	11	0.1990±0.0460	
Poor	12	0.1464±0.0316	
Tumor staging			0.014
IA–IB	14	0.2631±0.0319	
IIA–IIB	17	0.1513±0.0321	
IIIA	6	0.1091±0.0253	

MIIP, migration and invasion inhibitor protein; SE, standard error of the mean.

**Table III:** Correlation between MIIP3 protein expression and various clinicopathological parameters.

		MIIP expression		
			
Clinicopathological feature	No. of patients	Positive (n=36)	Negative (n=58)	P-value
Gender				0.250
Male	54	18	36	
Female	40	18	22	
Age				0.133
<60	43	20	23	
≥60	51	16	35	
Pathology				0.014
Adenocarcinoma	45	23	22	
Squamous cell carcinoma	49	13	36	
Differentiation status				0.680
Well	29	13	16	
Moderate	43	15	28	
Poor	22	8	14	
Tumor staging				0.002
IA–IB	30	18	12	
IIA–IIB	45	16	29	
IIIA	19	2	17	

MIIP, migration and invasion inhibitor protein.

**Table IV:** Univariate and multivariate analyses of MIIP protein expression with regard to overall survival.

	Overall survival		
	
Variables	HR^a^	95% CI^b^	P-value
Univariate analysis		0.337–0.954	0.033
Negative expression	1.000		
Positive expression	0.567		
Multivariate analysis		0.205–0.963	0.040
Negative expression	1.000		
Positive expression	0.444		

a HR was estimated using the Cox proportional-hazards regression model;

b CI of the estimated HR.

Multivariate models were adjusted for patient gender, age, pathology, differentiation status and tumor stage. HR, hazard ratio; CI, confidence interval; MIIP, migration and invasion inhibitor protein.
